# Supplementary material for: ARID1A mutations confer intrinsic and acquired resistance to cetuximab treatment in colorectal cancer
Source: Nat Commun. 2022 Sep 19;13:5478. doi: 10.1038/s41467-022-33172-5 (PMC9482920; doi:10.1038/s41467-022-33172-5)
Supplement: Supplementary file 1 — Supplementary Information [file 41467_2022_33172_MOESM1_ESM.pdf]

Supplementary information

Supplement to Johnson *et al*. "ARID1A mutations induce an EGFR-like signature and confer intrinsic and acquired resistance to cetuximab treatment in colorectal cancer"

Table of Contents

Supplemental Figures *2*

Supplemental Figure 1 3

Supplemental Figure 2 4

Supplemental Figure 3 5

Supplemental Figure 4 6

Supplemental Figure 5 7

Supplemental Figure 6 8

Supplemental Figure 7 9

Supplemental Figure 8 10

Supplemental Figure 9 11

Supplemental Figure 10 12

Supplemental Figure 11 13

Supplemental Figure 12 14

Supplemental Figure 13 15

Supplemental Figure 14 16

Supplemental Figure 15 17

Supplemental Figure 16 18

Supplementary Tables *19*

Supplementary Table 1 19

Supplementary Table 2 20

Supplemental Figures

Supplemental Figure 1

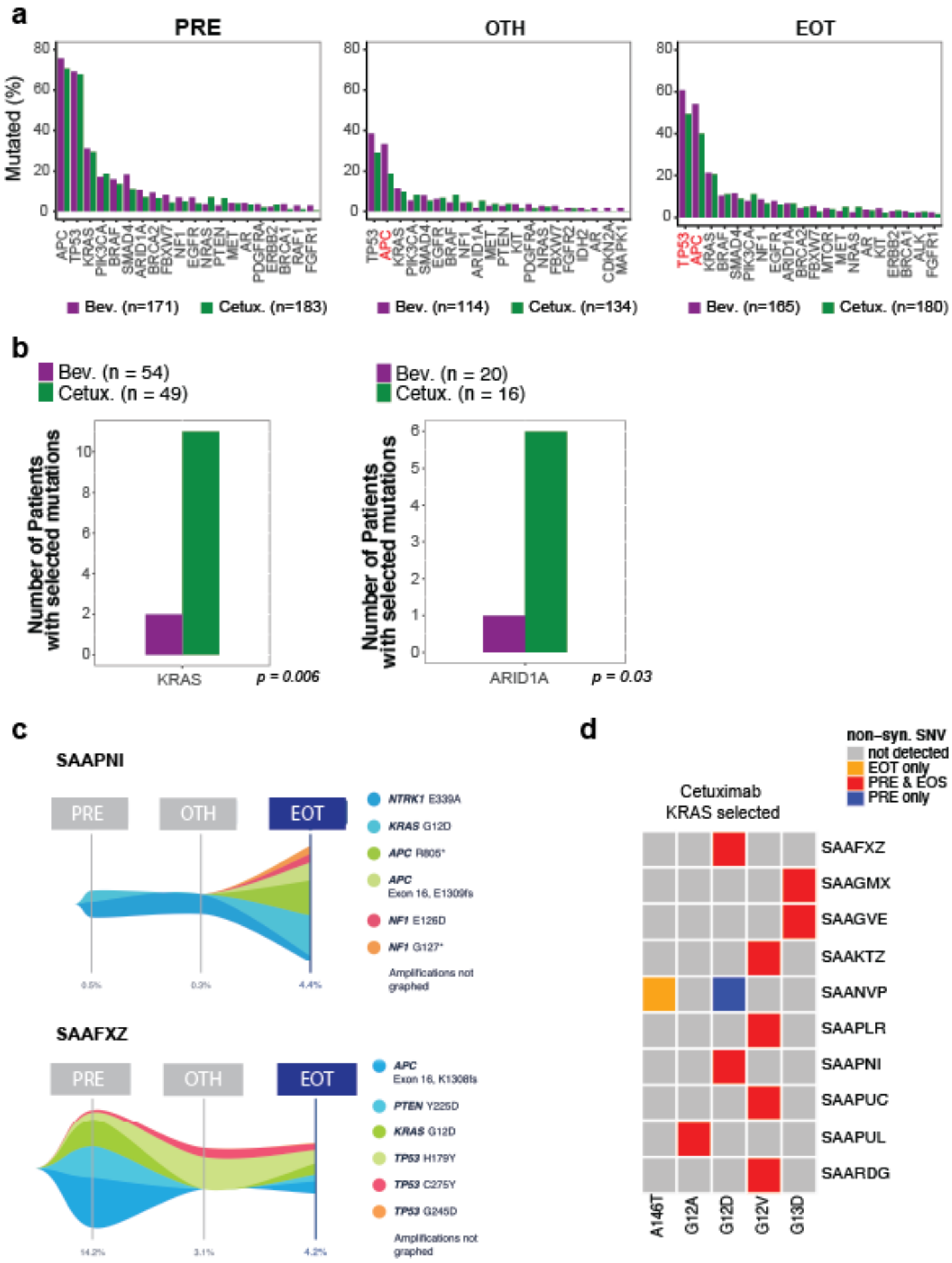

Supplemental Fig. 1. Summary of mutations detected in cfDNA from CALGB/SWOG 80405 liquid biopsies. a) Genomic alterations detected in cfDNA of 1L mCRC patients in the CALGB/SWOG 80405 trial at baseline (*left*; PRE), on treatment (*middle*; OTH) and at the end of study (*right*; EOT). Genomic alterations include non-synonymous single nucleotide variants (SNV), indels, and gene rearrangements. (b) Number of patients with selected alterations in KRAS (*top*) and ARID1A (*bottom*) by treatment group. Numbers between parentheses indicate the total number of patients with an alteration in the respective gene at baseline and/or end of study. Two-tailed Fisher's exact test with adjusted p values by Benjamini-Hochberg method shown. (c) Representative mutation evolution maps for cetuximab-treated patients with selected KRAS mutations. The maximum observed % MAF is shown at the bottom for each timepoint. PRE, at baseline; OTH, on treatment; EOT, at progression or end of study. (d) Overview of non-synonymous SNVs detected in KRAS, in cetuximab-treated patients with selected KRAS mutations.

Supplemental Figure 2

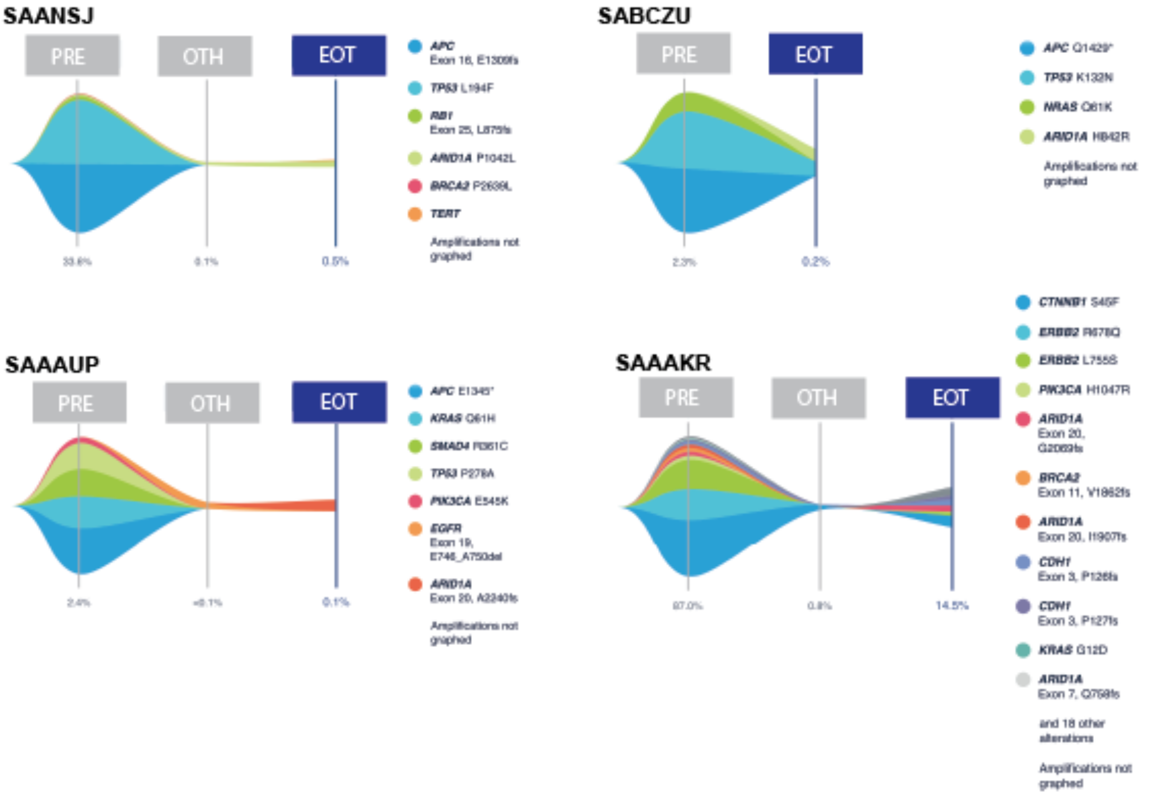

Supplemental Fig. 2. Representative cfDNA mutation evolution maps with selected ARID1A mutations. (a) Mutation evolution maps for four cetuximab-treated patients with selected ARID1A mutations. The maximum observed % MAF is shown at the bottom for each timepoint. PRE, at baseline; OTH, on treatment; EOT, at progression or end of study.

Supplemental Figure 3

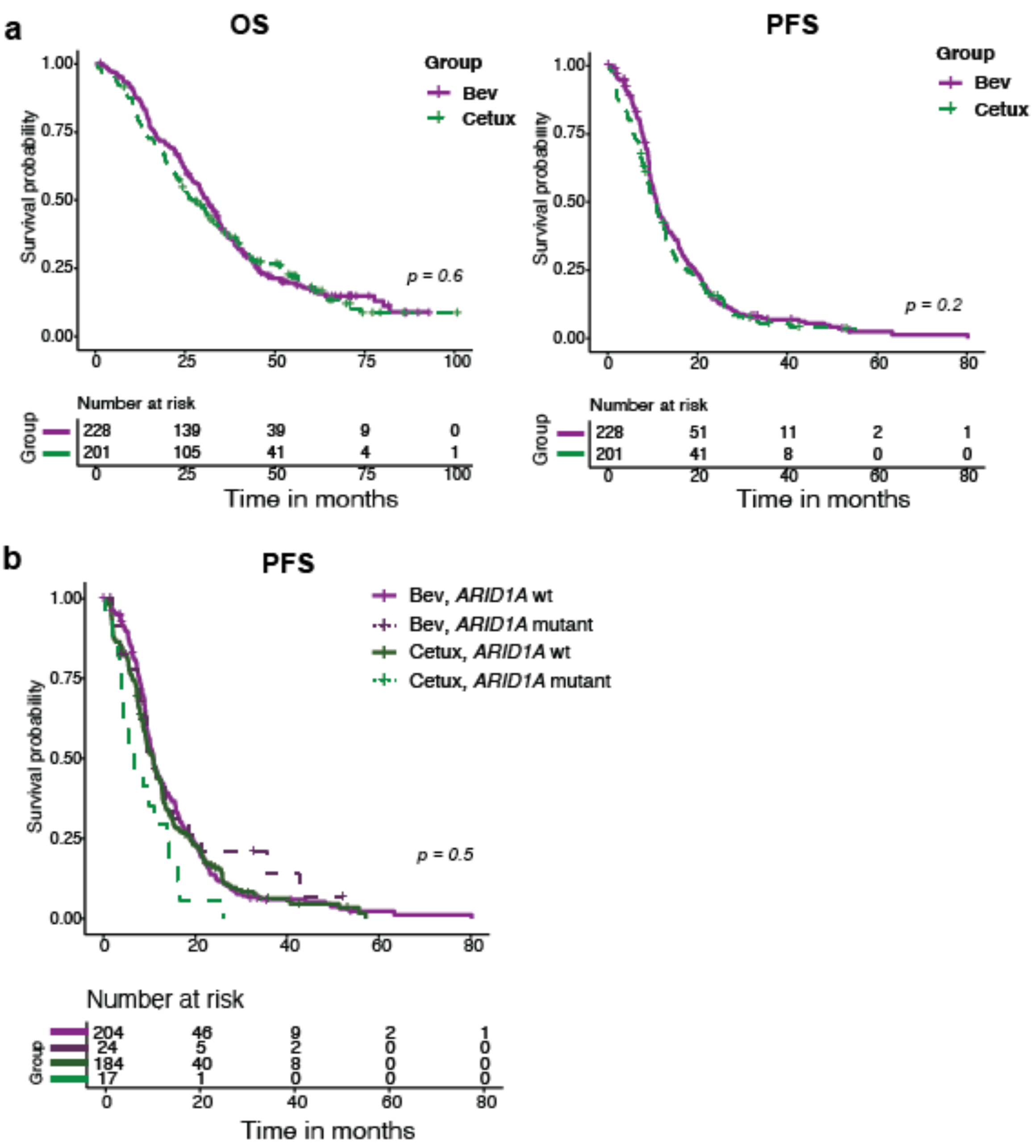

Supplemental Fig. 3. ITT vs BEP survival curves. (a) Kaplan-Meier curves showing OS (*left*) and PFS (*right)* for the CALGB/SWOG 80405 biomarker evaluable population (BEP) stratified by treatment arm with log-rank p values shown. BEP is defined as patients with archival tissue available for targeted sequencing. (b) Kaplan-Meier curves showing PFS for 429 CALGB/SWOG 80405 patients with archival tissue available for targeted sequencing, stratified by ARIDA mutation status and treatment arm with log-rank p values shown. Patients with ARID1A alterations classified as known or likely were considered mutant. Source data are provided as a Source Data file.

Supplemental Figure 4

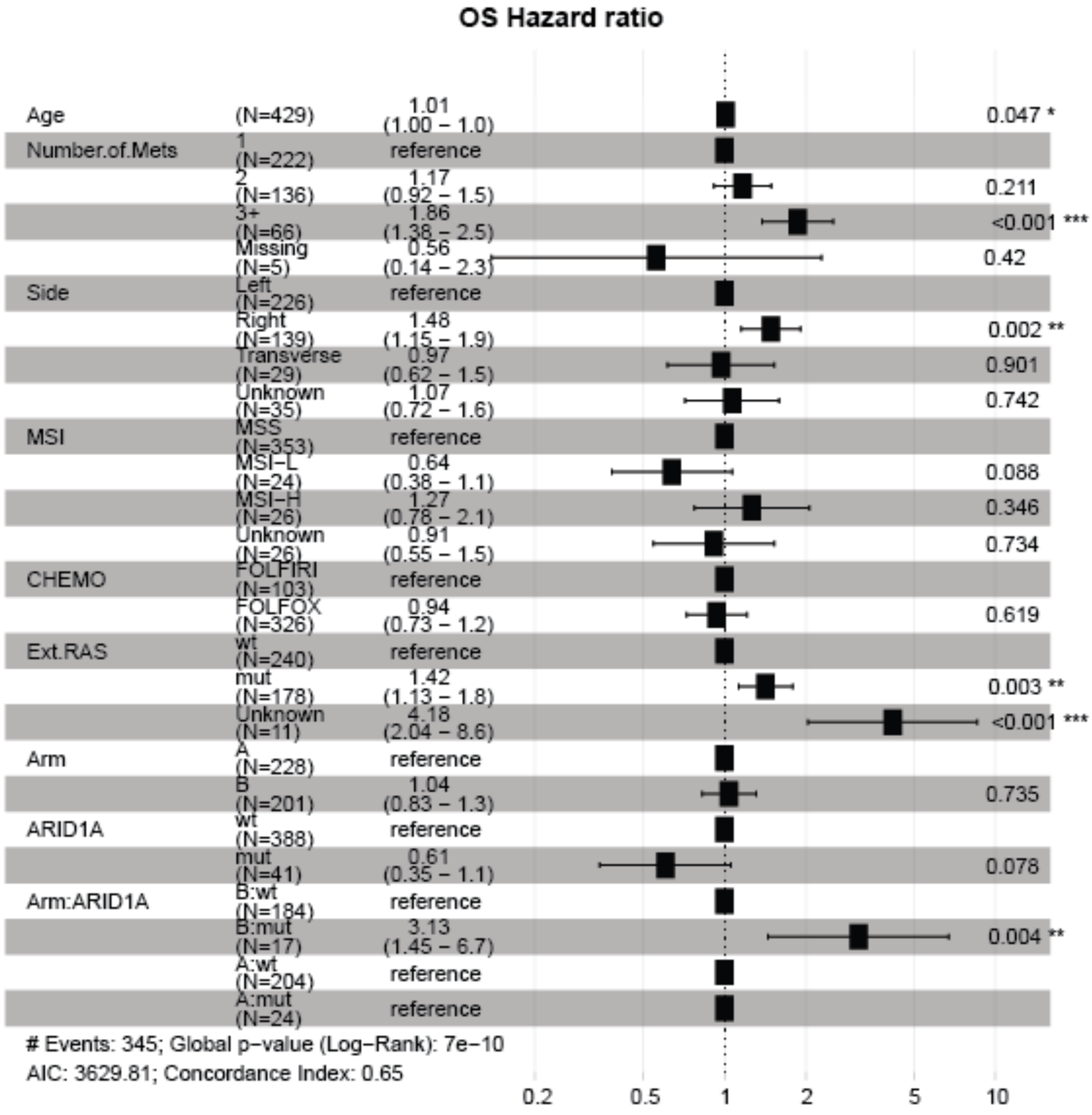

Supplemental Fig. 4. Multivariable model for overall survival with adjustment for established clinical variables and chemo protocol. Forest plots showing multivariate Cox proportional hazard ratios with 95% confidence intervals and P-values for age, number of metastases (mets), tumor side, MSI status, chemo protocol, treatment arm, ARID1A mutation status, and the interaction between treatment arm and ARID1A mutation status. Arm A corresponds to bevacizumab-treated patients and Arm B to cetuximab-treated patients. Solid square shows hazard ratio with horizontal bars showing 95% confidence intervals, vertical line corresponds to hazard ratio = 1.0. Source data are provided as a Source Data file

Supplemental Figure 5

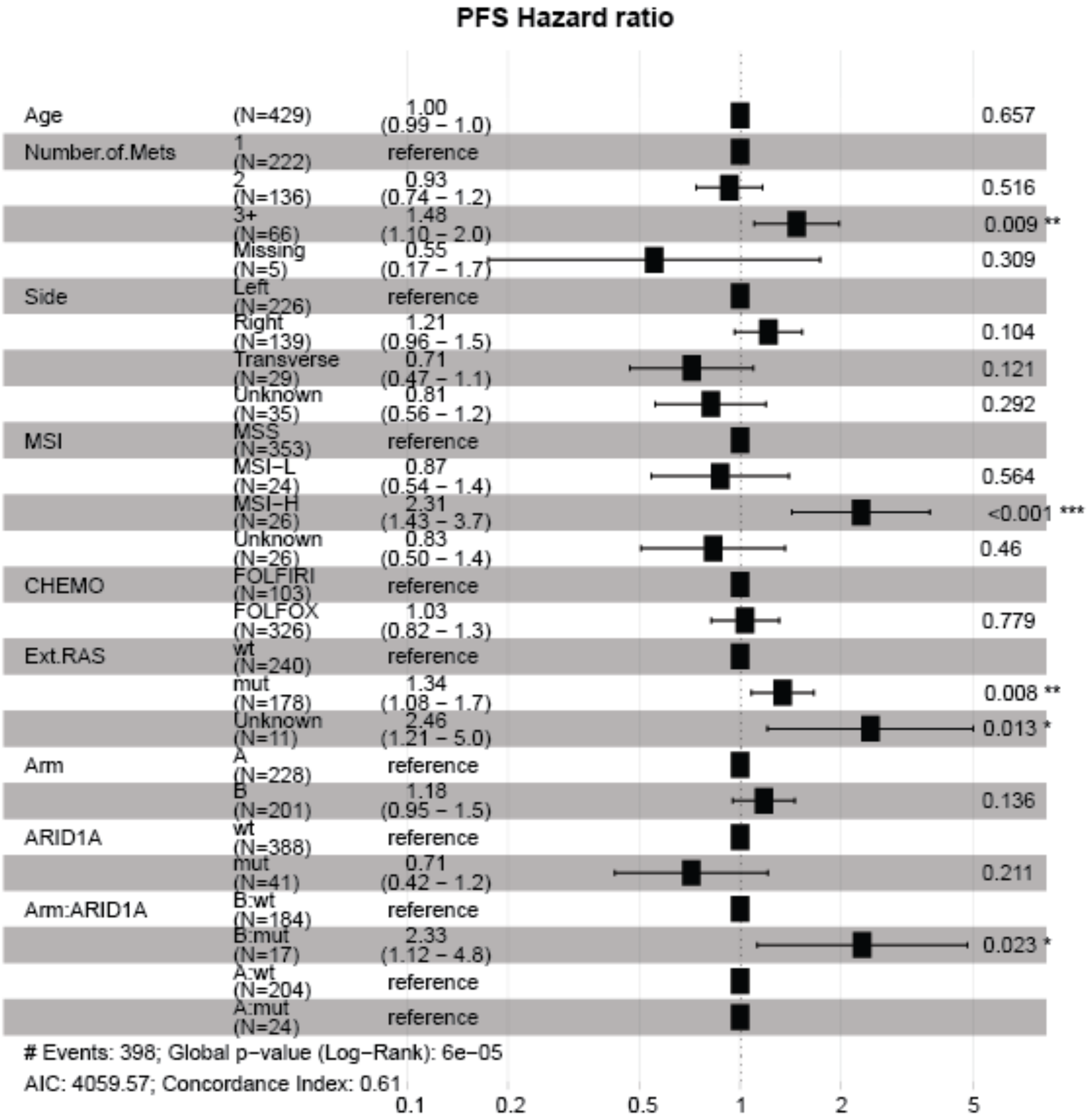

Supplemental Fig. 5. Multivariable model for progression-free survival with adjustment for established clinical variables and chemo protocol. Forest plots showing multivariate Cox proportional hazard ratios with 95% confidence intervals and P-values for age, number of mets, tumor side, MSI status, chemo protocol, treatment arm, ARID1A mutation status, and the interaction between treatment arm and ARID1A mutation status. Arm A corresponds to bevacizumab-treated patients and Arm B to cetuximab-treated patients. Solid square shows hazard ratio with horizontal bars showing 95% confidence intervals, vertical line corresponds to hazard ratio = 1.0.

Supplemental Figure 6

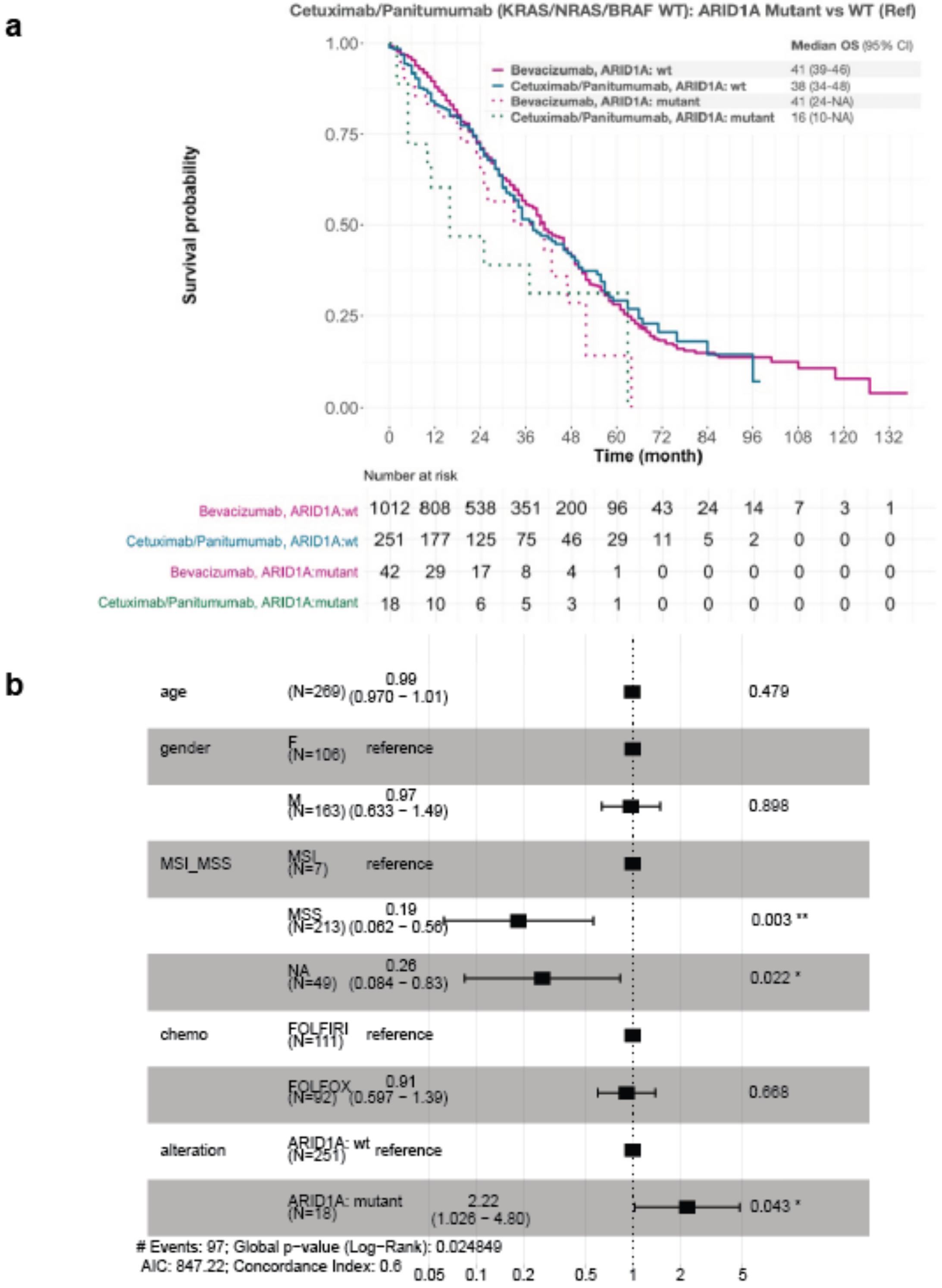

Supplemental Fig. 6. Real world data multivariable model for overall survival with adjustment for established clinical variables and chemo protocol. Real world data is from the Flatiron Health-Foundation Medicine CRC clinico-genomic database. (a) Kaplan-Meier curve showing OS for patients

treated with cetuximab or panitumumab (anti-EGFR) and bevacizumab (anti-VEGF) in 1L therapy with table (*top right)* of unadjusted OS median with confidence intervals for each group. (b) Among patients receiving cetuximab or panitumumab and who were also wildtype for KRAS, NRAS and BRAF, forest plots of HRs for ARID1A mutation status and other factors (age, gender, MSI status, chemo protocol) from a multivariate Cox PH model is shown. Solid square shows hazard ratio with horizontal bars showing 95% confidence intervals, vertical line corresponds to hazard ratio = 1.0.

Supplemental Figure 7

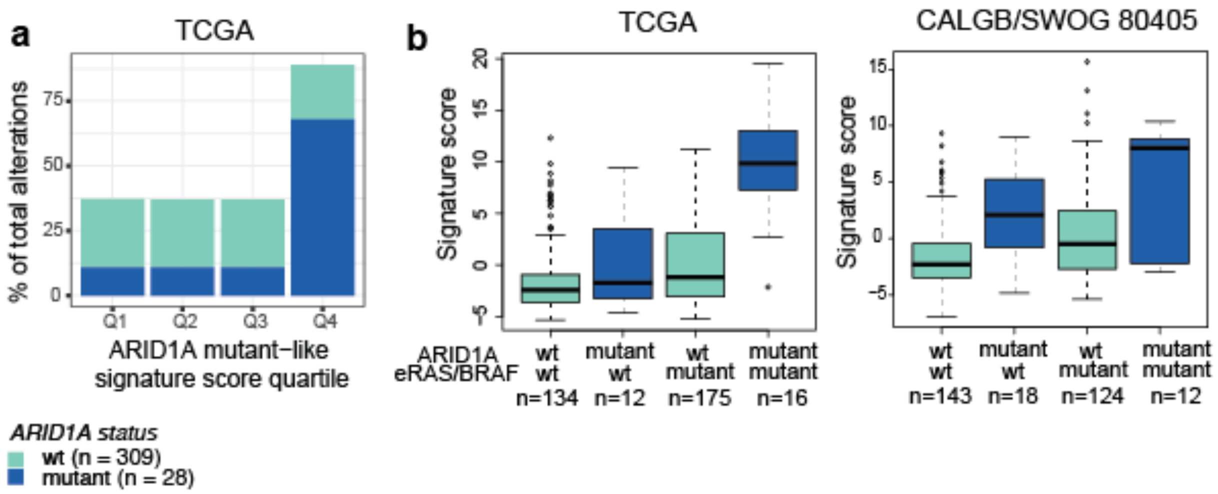

Supplemental Fig. 7. ARID1A mutant-like signature scores by *ARID1A* and eRAS/BRAF mutation status. ARID1A mutant-like signature enriches for patients with defective components of the SWI/SNF complex. (a) ARID1A mutant-like signature score split into quartiles with the % of alterations in each quartile that were ARID1A mutant and WT shown for TCGA cohort. (b) Distribution of ARID1A signature score by ARID1A and eRAS/BRAF status in TCGA patients. The interquartile range (IQR) is depicted by the box with the median represented by the center line. Whiskers maximally extend to 1.5 × IQR (with outliers shown). Source data are provided as a Source Data file.

Supplemental Figure 8

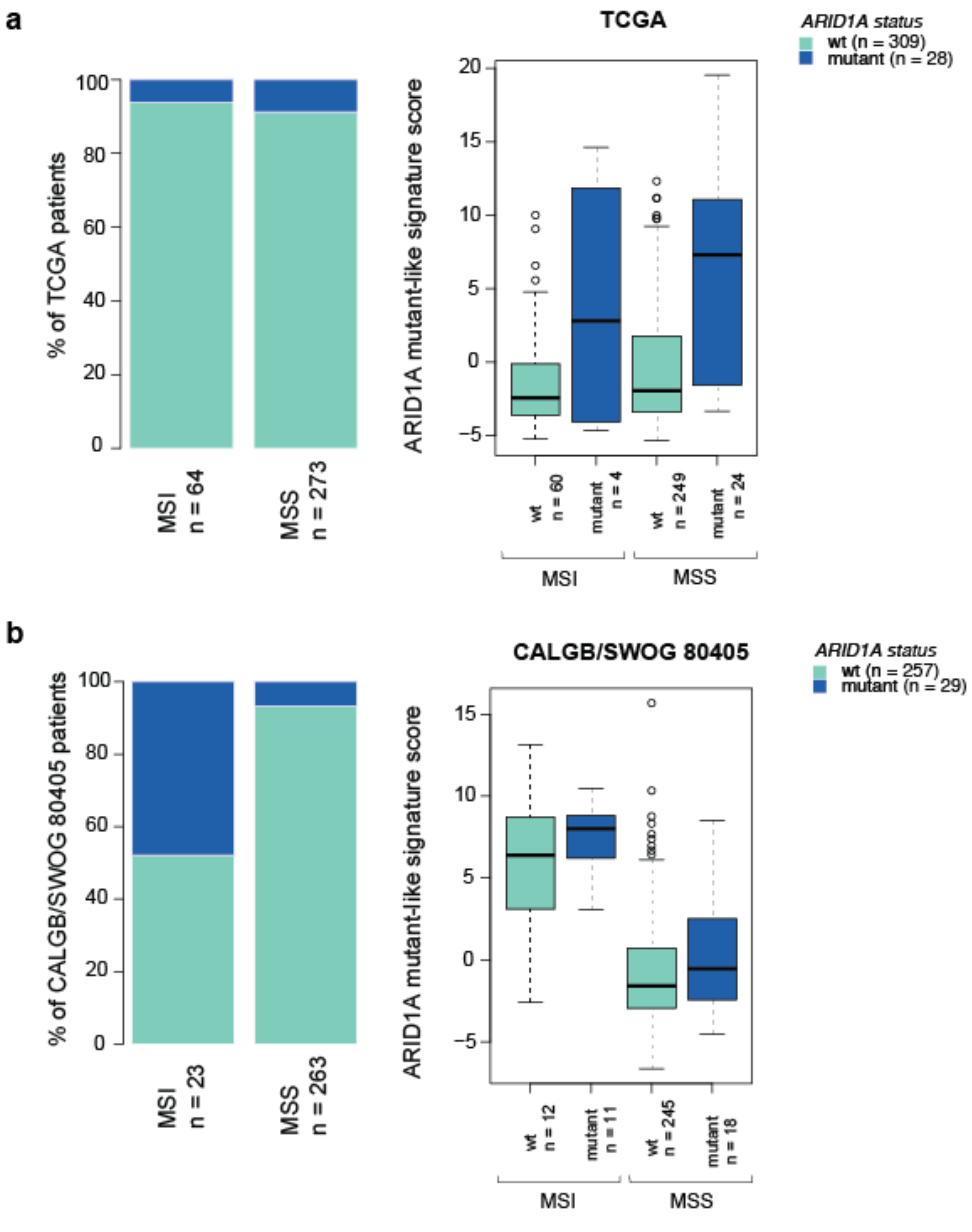

Supplemental Fig. 8. ARID1A mutant-like signature scores by MSI status in TCGA (a) and CALGB/SWOG 80405 (b). The proportion of ARID1A WT and mutant tumors in MSI and MSS tumors (*left*) and ARID1A mutant-like signature scores stratified by ARID1A mutation and MSI status (*right*).

The interquartile range (IQR) is depicted by the box with the median represented by the center line. Whiskers maximally extend to 1.5 × IQR (with outliers shown). Source data are provided as a Source Data file.

Supplemental Figure 9

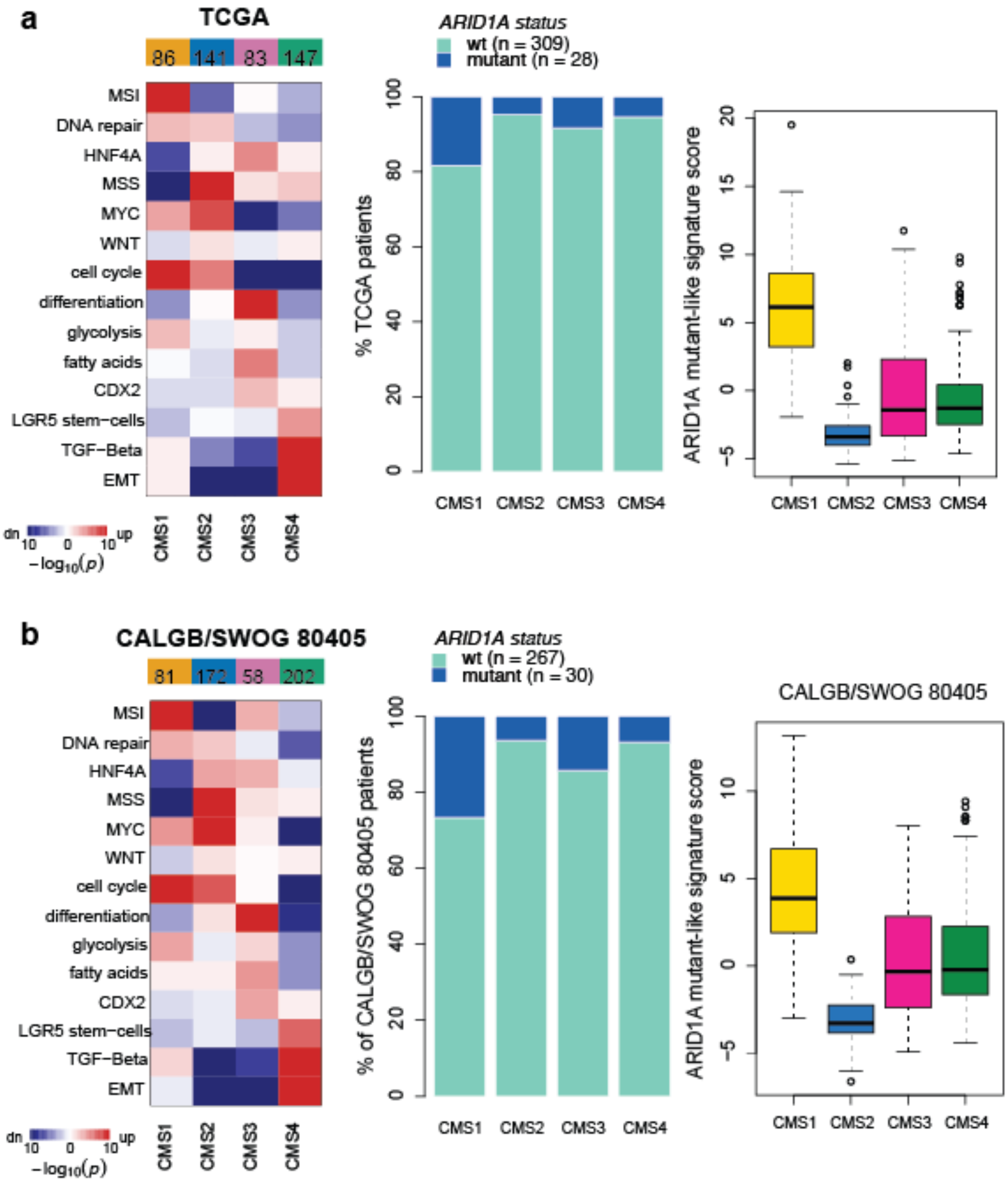

Supplemental Fig. 9. ARID1A mutant-like signature scores by CMS subtype in TCGA (a) and CALGB/SWOG 80405 (b). Heatmap shows results from CMScaller mRNA gene set analysis,

confirming enrichment of known characteristics in each CMS group (*left).* The proportion of ARID1A WT and mutant tumors in MSI and MSS tumors (*middle*) and ARID1A mutant-like signature scores stratified by ARID1A mutation and MSI status (*right*). The interquartile range (IQR) is depicted by the box with the median represented by the center line. Whiskers maximally extend to 1.5 × IQR (with outliers shown). Source data are provided as a Source Data file.

Supplemental Figure 10

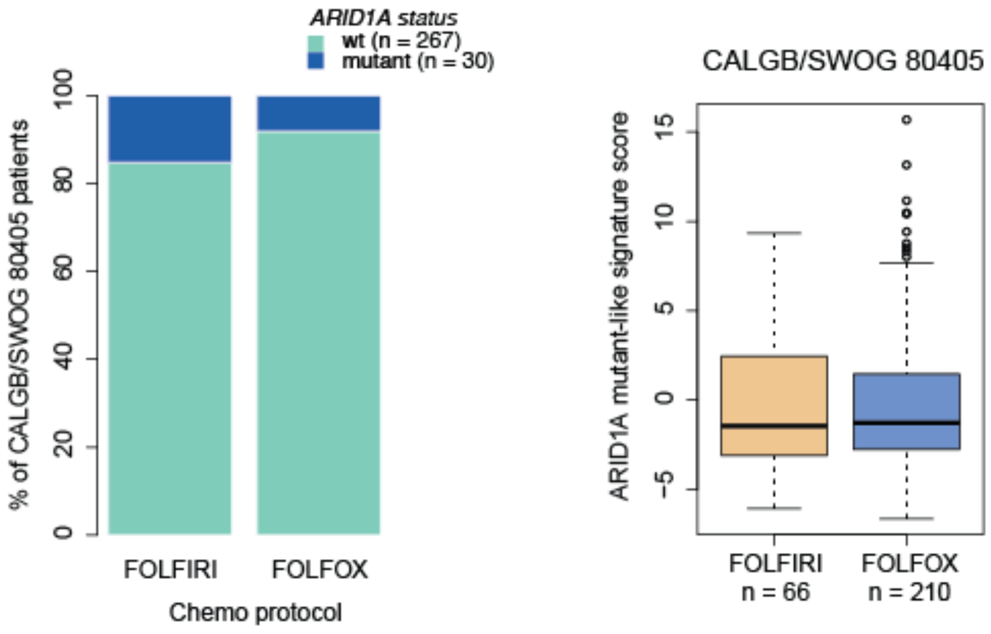

Supplemental Fig. 10. ARID1A mutant-like signature scores by chemo protocol in CALGB/SWOG 80405. The proportion of ARID1A WT and mutant tumors in FOLFOX and FOLFIRI treated groups (*left*) and ARID1A mutant-like signature scores stratified by chemo protocol used (*right*). The interquartile range (IQR) is depicted by the box with the median represented by the center line. Whiskers maximally extend to 1.5 × IQR (with outliers shown). Source data are provided as a Source Data file.

Supplemental Figure 11

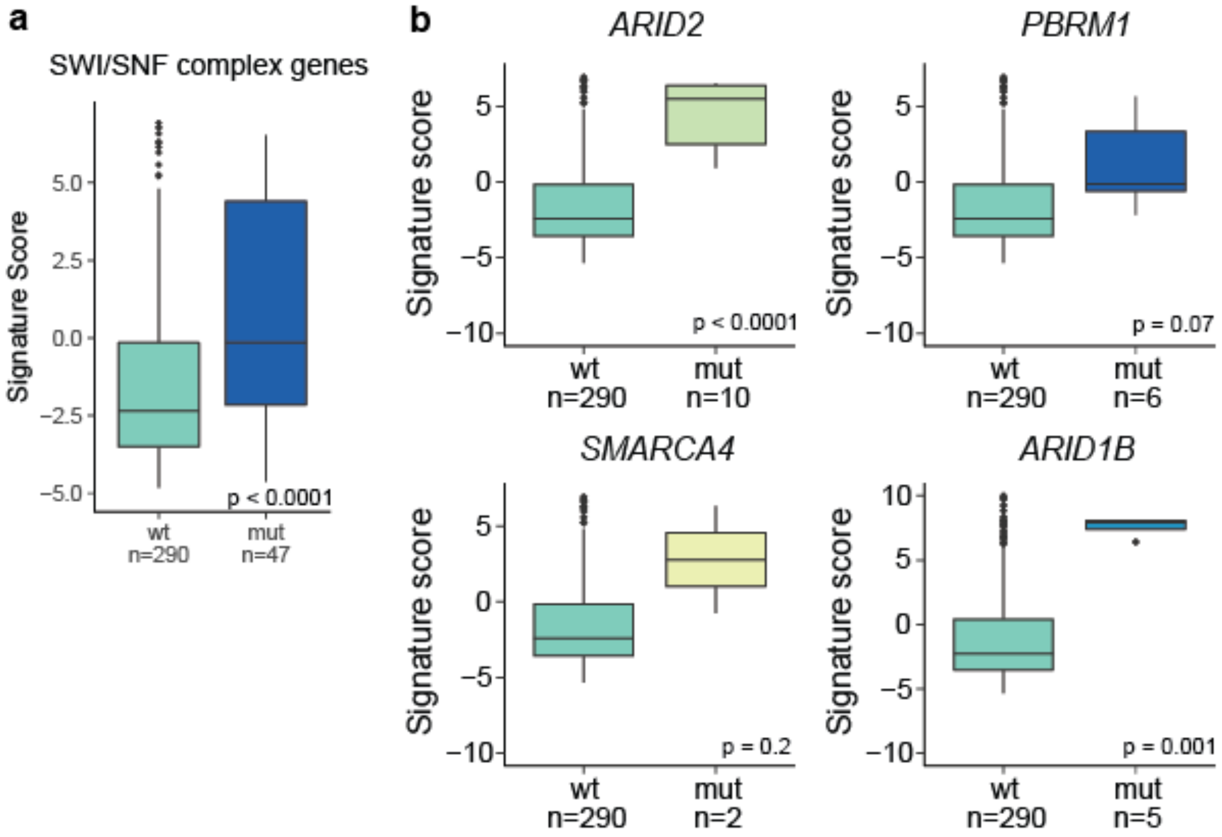

Supplemental Fig. 11. ARID1A mutant-like signature scores by SWI/SNF complex (*ARID2, PBRM1, SMARCA4, ARID1B*) mutation status. ARID1A mutant-like signature enriches for patients with defective components of the SWI/SNF complex. (a, b) Distribution of ARID1A signature score by the mutation status of SWI/SNF complex subunits (a) including ARID1A, ARID2, PBRM1, SMARCA4, ARID1B and of each individual member (b) measured in TCGA. Wildtype tumors lack an alteration in any SWI/SNF complex member. P values determined by two-sided Wilcoxon rank sum test. Alterations annotated based on selected functional events (SFE) reported[1]. The interquartile range (IQR) is depicted by the box with the median represented by the center line. Whiskers maximally extend to 1.5 × IQR (with outliers shown).

Supplemental Figure 12

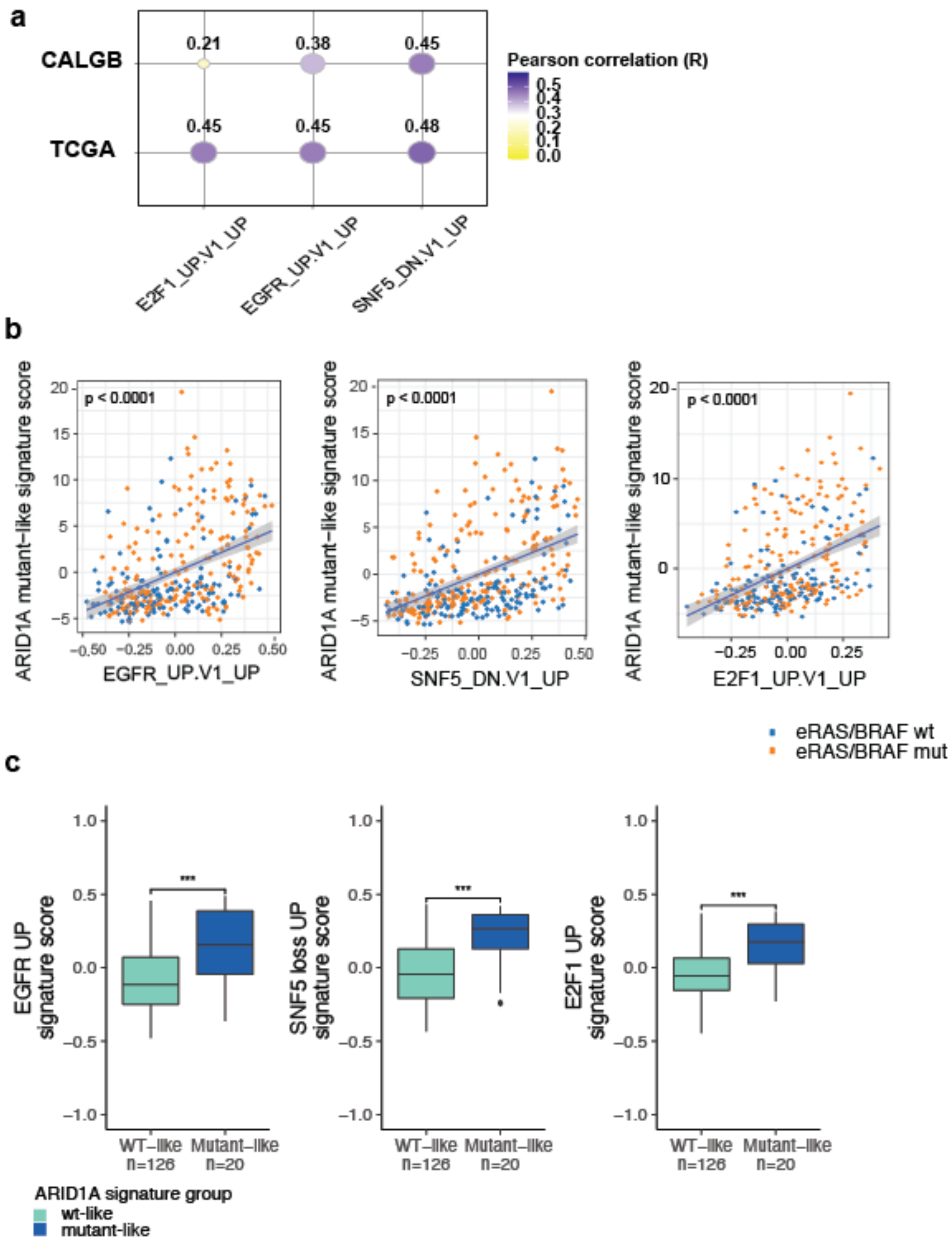

Supplemental Fig. 12. ARID1A mutant-like signature relationship with know oncogenic signatures. (a) Overview of C6 oncogenic signatures that correlate with the ARID1A signature in the TCGA cohort, with absolute $R^2 > 0.4$ by Pearson's correlation test. (b) Pearson's correlation between ARID1A mutant-like signature scores and MSigDB C6 oncogenic signature GSVA scores with $R > 0.4$. P value derived from two-sided Pearson's product-moment correlation test as implemented in the cor.test() function in R against the null hypothesis correlation is equal to 0. Error bands represent 95% confidence level interval for the linear model predictions. (c) C6 oncogenic signature scores stratified by ARID1A WT-like and mutant-like grouping. P values determined by two-sided Wilcoxon rank sum test (NS, no statistical significance; *$P < 0.05$, **, $P < 0.01$; *** $P < 0.001$). The interquartile range (IQR) is depicted by the box with the median represented by the center line. Whiskers maximally extend to 1.5 × IQR (with outliers shown). Source data are provided as a Source Data file.

Supplemental Figure 13

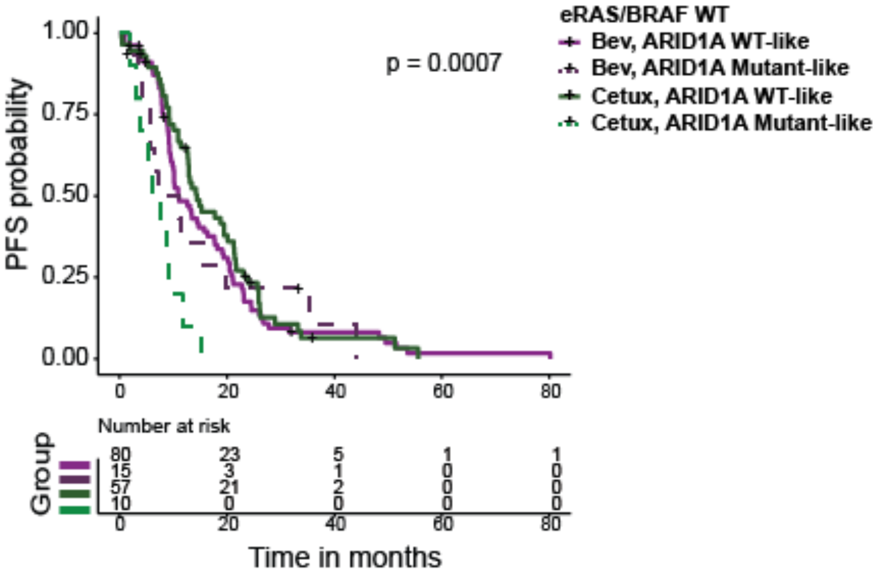

Supplemental Fig. 13. Progression free survival stratified by treatment arm and ARID1A mutant-like signature status. Kaplan-Meier curves showing PFS in n = 162 eRAS/BRAF WT patients stratified by ARID1A mutant-like signature group and treatment arm with *P* values from log-rank test shown.

Supplemental Figure 14

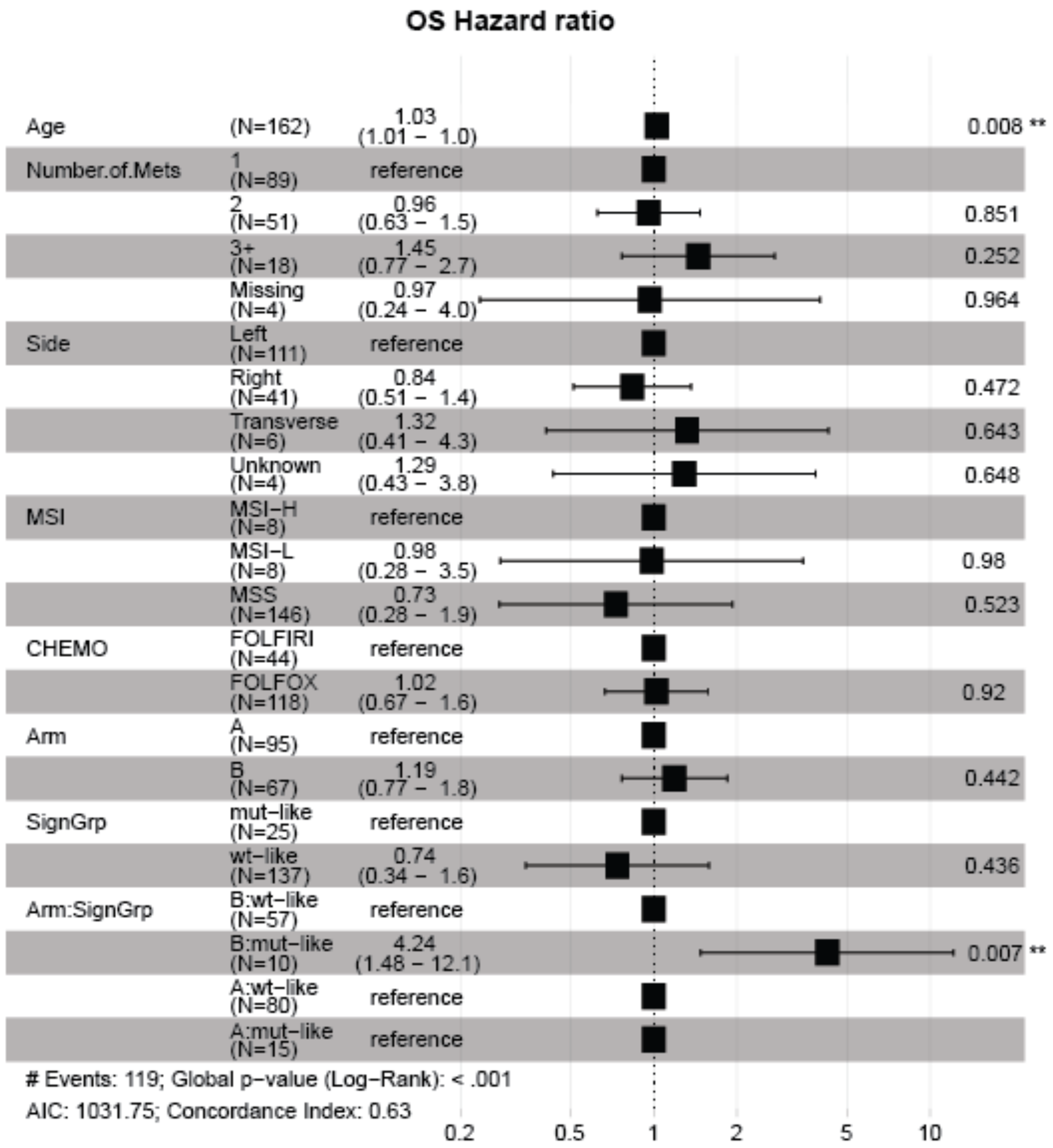

Supplemental Fig. 14. Multivariable model for overall survival with adjustment for established clinical variables and chemo protocol. Forest plots showing multivariate Cox proportional hazard ratios with 95% confidence intervals and P-values for age, number of mets, tumor side, MSI status, chemo protocol, treatment arm, ARID1A mutant-like signature group, and the interaction between treatment arm and ARID1A mutant-like signature group. Arm A corresponds to bevacizumab-treated patients and Arm B to cetuximab-treated patients. Solid square shows hazard ratio with horizontal bars showing 95% confidence intervals, vertical line corresponds to hazard ratio = 1.0.

Supplemental Figure 15

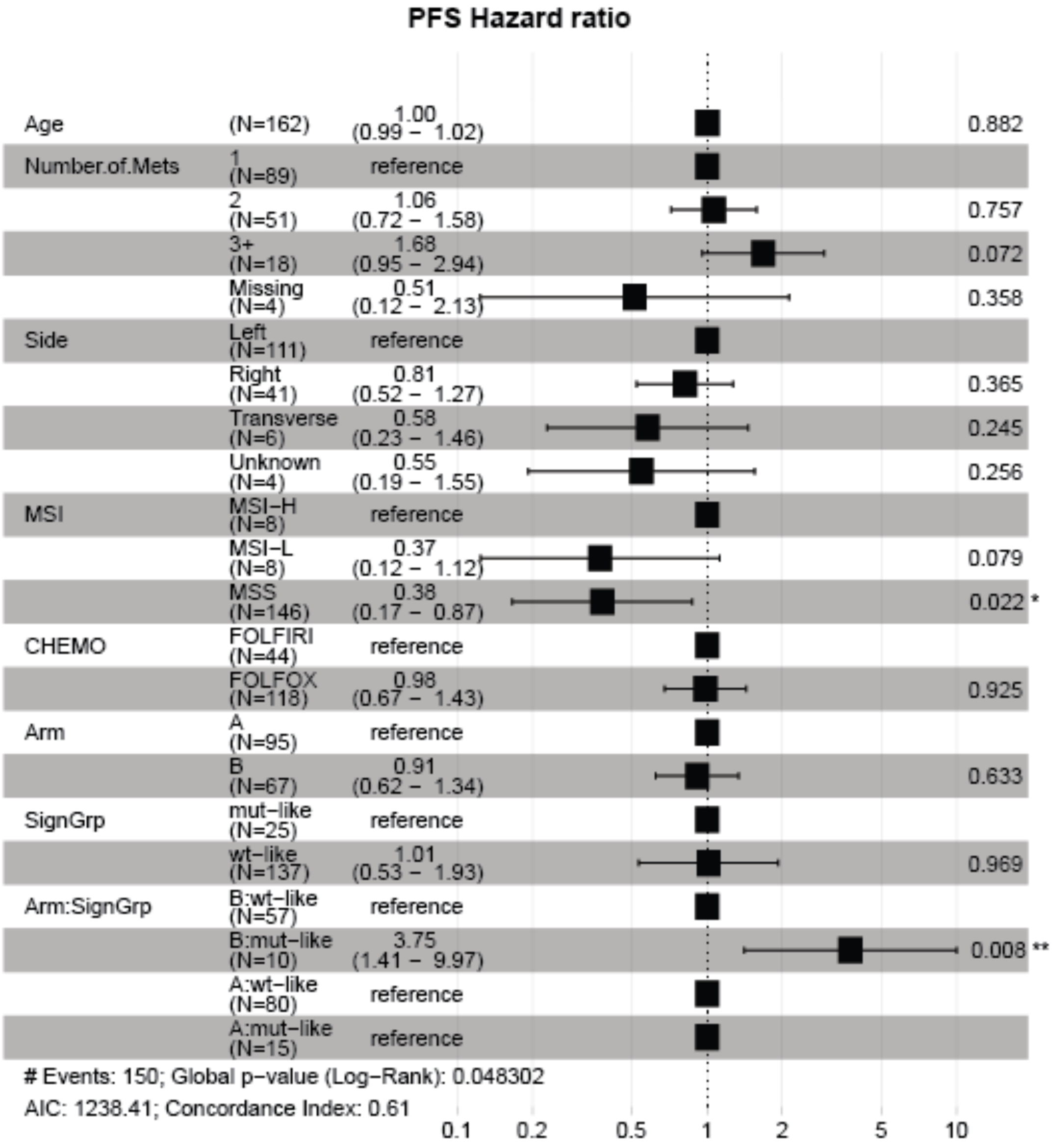

Supplemental Fig. 15. Multivariable model for progression-free survival with adjustment for established clinical variables and chemo protocol. Forest plots showing multivariate Cox proportional hazard ratios with 95% confidence intervals and P-values for age, number of mets, tumor side, MSI status, chemo protocol, treatment arm, ARID1A mutant-like signature group, and the interaction between treatment arm and ARID1A mutant-like signature group. Arm A corresponds to bevacizumab-treated patients and Arm B to cetuximab-treated patients. Solid square shows hazard ratio with horizontal bars showing 95% confidence intervals, vertical line corresponds to hazard ratio = 1.0.

Supplemental Figure 16

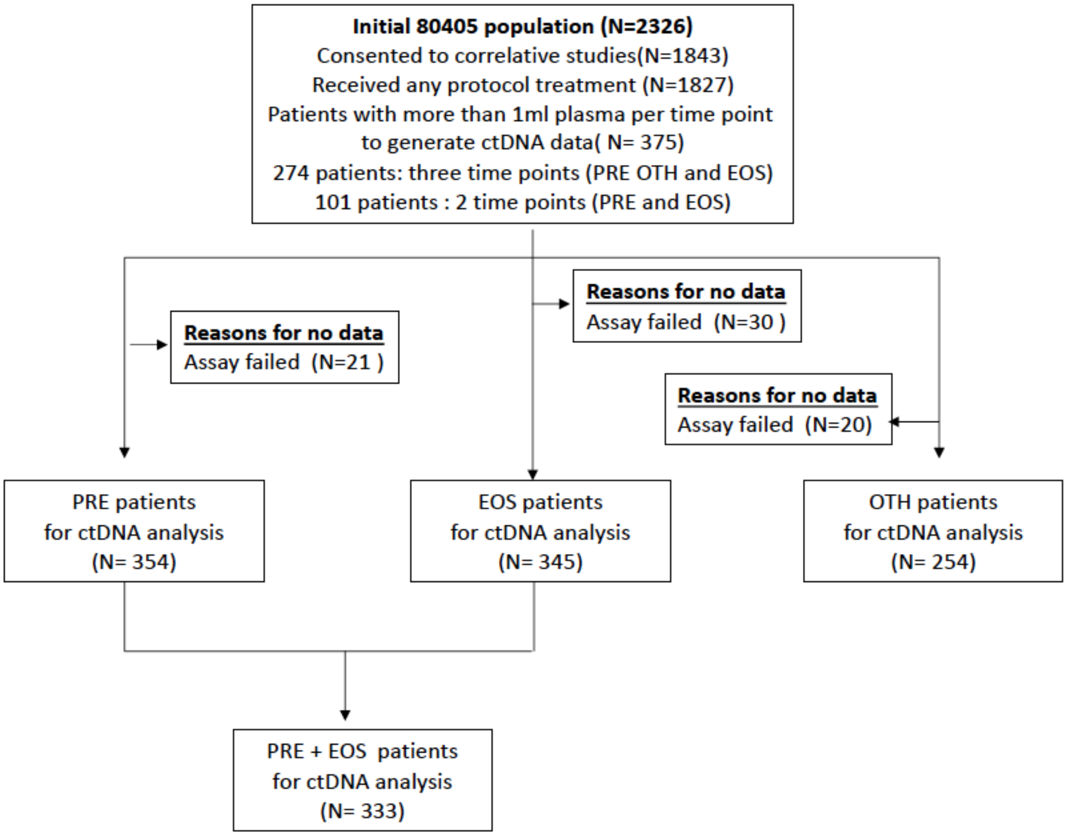

Supplemental Fig. 16. Flow chart of patients enrolled and liquid biopsies collected at each timepoint for CALGB/SWOG 80405 study. Abbreviations: ctDNA, cell-free tumor DNA; PRE, at baseline; OTH, on treatment; EOT, at progression or end of study

Supplementary Tables

Supplementary Table 1

	Treatment	Chemo_ protocol	Best response	Progression date	PFS Status	PFS (days)	PRE	OTX	EOS	Days Bw PRE and OTX	Days bw PRE and EOS
SAANDA	Cetuximab	FOLFIRI	NA	6/25/07	Event	96	3/26/07	5/22/07	7/3/07	57	99
SAARSX	Cetuximab	FOLFOX	SD	8/23/10	Event	178	3/3/10	5/5/10	7/12/10	63	131
SAASGM	Cetuximab	FOLFIRI	PR	10/2/13	Event	813	7/13/11	9/7/11	10/25/12	56	470
SABCZU	Cetuximab	FOLFOX	SD	4/24/11	Event	153	11/30/10	NA	4/26/11	NA	147
SAANSJ	Cetuximab	FOLFOX	PR	9/16/08	Event	788	1/7/08	3/18/10	3/22/10	801	805
SAAAUP	Cetuximab	FOLFOX	PR	4/2/12	Event	333	5/5/11	7/14/11	11/3/11	70	182
SAAAKR	Bevacizumab	FOLFIRI	SD	6/6/12	Event	176	12/13/11	4/3/12	6/19/12	111	188

Supplementary Table 1. Abbreviations: PRE, at baseline; OTH, on treatment; EOT, at progression or end of study.

Supplementary Table 2

Prognositic Variable	Arm A ITT	Arm A BEP	Arm B ITT	Arm B BEP
**Age**				
N	502	228	523	201
Mean	59.5	59.23	59.4	58.78
Median	60.28	59.59	59.67	59.09
Min-Max	23.17 - 85.01	23.17 - 80.19	20.77 - 89.51	20.77 - 84.49
**Gender**				
Total (non-NA)	502	228	523	201
Female	191 (38.05%)	87 (38.16%)	199 (38.05%)	76 (37.81%)
Male	311 (61.95%)	141 (61.84%)	324 (61.95%)	125 (62.19%)
**Race**				
Total (non-NA)	501	228	520	198
NA's	< 5	0	<5	<5
African American	55 (10.98%)	23 (10.09%)	54 (10.38%)	24 (12.12%)
American Indian	3 (0.6%)	0 (0%)	< 5 (0.58%)	<5 (1.01%)
or Alaska Native				
Asian	6 (1.2%)	3 (1.32%)	14 (2.69%)	< 5 (1.52%)
Native Hawaiian	0 (0%)	0 (0%)	< 5 (0.38%)	< 5 (0.51%)
or Pacific Islander				
Not Reported	1 (0.2%)	1 (0.44%)	0 (0%)	0 (0%)
Unknown	1 (0.2%)	1 (0.44%)	7 (1.35%)	3 (1.52%)
White	435 (86.83%)	200 (87.72%)	440 (84.62%)	165 (83.33%)
**Synchronous vs Metachronous**				
Total (non-NA)	487	226	512	199
NA's	15	< 5	11	< 5
Metachronous	103 (21.15%)	38 (16.81%)	116 (22.66%)	44 (22.11%)
Synchronous	384 (78.85%)	188 (83.19%)	396 (77.34%)	155 (77.89%)
**ECOG.PS**				
Total (non-NA)	502	228	523	201
0	301 (59.96%)	131 (57.46%)	309 (59.08%)	119 (59.2%)
1	199 (39.64%)	97 (42.54%)	214 (40.92%)	82 (40.8%)
2	< 5 (0.4%)	0 (0%)	0 (0%)	0 (0%)
**SWOG KRAS Status**				
Total (non-NA)	500	228	522	200
NA's	< 5	0	< 5	<5
Mutant	104 (20.8%)	37 (16.23%)	103 (19.73%)	40 (20%)
Undetermined	18 (3.6%)	1 (0.44%)	12 (2.3%)	2 (1%)
Wild Type	378 (75.6%)	190 (83.33%)	407 (77.97%)	158 (79%)
**Adjuvant Chemotherapy**				
Total (non-NA)	502	228	523	201
No	427 (85.06%)	203 (89.04%)	450 (86.04%)	175 (87.06%)
Yes	75 (14.94%)	25 (10.96%)	73 (13.96%)	26 (12.94%)
**Pelvic Radiation**				
Total (non-NA)	502	228	523	201
No	458 (91.24%)	214 (93.86%)	476 (91.01%)	188 (93.53%)
Yes	44 (8.76%)	14 (6.14%)	47 (8.99%)	13 (6.47%)

Supplementary Table 2. ITT vs BEP demographics. Abbreviations: ITT, Intention to Treat; BEP, Biomarker Evaluable Population.

References:

1 Mina, M. *et al.* Conditional Selection of Genomic Alterations Dictates Cancer Evolution and Oncogenic Dependencies. *Cancer Cell* **32**, 155-168 e156, doi:10.1016/j.ccell.2017.06.010 (2017).
