# Supplementary material for: ARID1A mutations confer intrinsic and acquired resistance to cetuximab treatment in colorectal cancer
Source: Nat Commun. 2022 Sep 19;13:5478. doi: 10.1038/s41467-022-33172-5 (PMC9482920; doi:10.1038/s41467-022-33172-5)
Supplement: Supplementary file 3 — Description of Additional Supplementary Files [file 41467_2022_33172_MOESM3_ESM.pdf]

Description of Additional Supplementary Files

File Name: Supplementary Data 1
Description: **Overview of genes with at least two selected mutations between baseline and end of study in either arm of the CALGB/SWOG 80405 trial.** Abbreviations: Bev, Bevacizumab; Cetux, Cetuximab.

File Name: Supplementary Data 2
Description: **List of ARID1A differentially expressed genes in TCGA cohort based on selected functional events (SFE) reported in Mina et al., 2017.**

File Name: Supplementary Data 3
Description: **Pathway commons enriched in top SELECT gene interactions with ARID1A in CRC.**

File Name: Supplementary Data 4
Description: **Pathway commons enriched in top SELECT gene interactions with ARID1A in lung cancer.**
