# Supplementary material for: ARID1A mutations confer intrinsic and acquired resistance to cetuximab treatment in colorectal cancer
Source: Nat Commun. 2022 Sep 19;13:5478. doi: 10.1038/s41467-022-33172-5 (PMC9482920; doi:10.1038/s41467-022-33172-5)
Supplement: Supplementary file 5 — Reporting Summary [file 41467_2022_33172_MOESM5_ESM.pdf]

nature portfolio

Corresponding author(s): Radia M. Johnson
Xueping Qu
Carlos Bais

Last updated by author(s): Sep 2, 2022

Reporting Summary

Nature Portfolio wishes to improve the reproducibility of the work that we publish. This form provides structure for consistency and transparency in reporting. For further information on Nature Portfolio policies, see our Editorial Policies and the Editorial Policy Checklist.

Statistics

For all statistical analyses, confirm that the following items are present in the figure legend, table legend, main text, or Methods section.

n/a	Confirmed	
☐	☒	The exact sample size (n) for each experimental group/condition, given as a discrete number and unit of measurement
☐	☒	A statement on whether measurements were taken from distinct samples or whether the same sample was measured repeatedly
☐	☒	The statistical test(s) used AND whether they are one- or two-sided<br>*Only common tests should be described solely by name; describe more complex techniques in the Methods section.*
☐	☒	A description of all covariates tested
☐	☒	A description of any assumptions or corrections, such as tests of normality and adjustment for multiple comparisons
☐	☒	A full description of the statistical parameters including central tendency (e.g. means) or other basic estimates (e.g. regression coefficient) AND variation (e.g. standard deviation) or associated estimates of uncertainty (e.g. confidence intervals)
☐	☒	For null hypothesis testing, the test statistic (e.g. F, t, r) with confidence intervals, effect sizes, degrees of freedom and P value noted<br>*Give P values as exact values whenever suitable.*
☒	☐	For Bayesian analysis, information on the choice of priors and Markov chain Monte Carlo settings
☒	☐	For hierarchical and complex designs, identification of the appropriate level for tests and full reporting of outcomes
☐	☒	Estimates of effect sizes (e.g. Cohen's d, Pearson's r), indicating how they were calculated

Our web collection on statistics for biologists contains articles on many of the points above.

Software and code

Policy information about availability of computer code

Data collection	No software was used to collect the data.
Data analysis	The SELECT algorithm was used to look for co-occuring and mutually exclusive mutations in FoundationCore database. A copy of the algorithm can be downloaded at http://ciriellolab.org/select/select.html at http://ciriellolab.org/select/select_1.0.tar.gz. RNAseq analysis was performed in R using limma (Version 3.53.5) bioconductor package. All statistical analysis was also run in R (version 4.2.0). Heatmaps were generated with ComplexHeatmap (version 2.13.0) bioconductor package. TCGA selected functional events used to annotate ARID1A and other SWI/SNF complex genes were obtained from http://ciriellolab.org/select/select.html and can be downloaded from http://ciriellolab.org/select/pancan23_dataset.zip. PDX GEO data was downloaded using the GEOquery (Version 2.65.2) bioconductor package with accession id GSE76402 and GPL annotation GPL10558 with the getGEO() function.

For manuscripts utilizing custom algorithms or software that are central to the research but not yet described in published literature, software must be made available to editors and reviewers. We strongly encourage code deposition in a community repository (e.g. GitHub). See the Nature Portfolio guidelines for submitting code & software for further information.

Data

Policy information about availability of data

All manuscripts must include a data availability statement. This statement should provide the following information, where applicable:

- Accession codes, unique identifiers, or web links for publicly available datasets
- A description of any restrictions on data availability
- For clinical datasets or third party data, please ensure that the statement adheres to our policy

The TCGA publicly available RNAseq data used in this study are available in the Firebrowse portal (version 2016 01 28) http://firebrowse.org/. TCGA gene expression

raw counts for colorectal cancer are available for download from https://gdac.broadinstitute.org/. The selected functional event mutation annotations for TCGA tumors are available from the Ciriello lab32 http://ciriellolab.org/select/pancan23_dataset.zip. The PDX gene expression data used in this study are available in the GEO database under accession code GSE7640258 [https://www.ncbi.nlm.nih.gov/geo/query/acc.cgi?acc=GSE76402]. The CALGB/SWOG 80405 RNAseq data previously generated and used in this study are available in the GEO database under accession code GSE196576 [https://www.ncbi.nlm.nih.gov/geo/query/acc.cgi?acc=GSE196576]. The CALGB/SWOG 80405 ctDNA data generated in this study have been deposited in the dbGAP database under accession code phs002941 [https://www.ncbi.nlm.nih.gov/projects/gap/cgi-bin/study.cgi?study_id=phs002941.v1.p1]. The data is available under restricted access due to them containing information that could compromise research participant privacy/consent. Access can be obtained by submitting a request to Xueping Qu (qu.xueping@gene.com). Individual-level data from the genotyped cohorts will be made available to researchers for academic purposes only following an approved analysis proposal for 1 year. Review timelines may vary but once approved access should be granted within a week. Real-world data in this study refers to observational data generated during routine clinical practice and collected outside regulated clinical trials by Flatiron Health and Foundation Medicine clinic-genomic database. Restrictions apply to the availability of the real-world data underlying the analysis for Foundation Medicine, Inc and Flatiron Health-Foundation Medicine CRC clinico-genomic database. For further details on Roche's Global Policy on the Sharing of Clinical Information, and how to request access to related clinical study documents, see https://www.roche.com/research_and_development/who_we_are_how_we_work/clinical_trials/our_commitment_to_data_sharing.htm. The remaining data are available within the Article, Supplementary Information or Source Data file.

Field-specific reporting

Please select the one below that is the best fit for your research. If you are not sure, read the appropriate sections before making your selection.

[x] Life sciences   [ ] Behavioural & social sciences   [ ] Ecological, evolutionary & environmental sciences

For a reference copy of the document with all sections, see nature.com/documents/nr-reporting-summary-flat.pdf

Life sciences study design

All studies must disclose on these points even when the disclosure is negative.

Sample size	Sample size was determined per the original clinical trial (https://jamanetwork.com/journals/jama/fullarticle/2632502) and further subset on patients with biomarker data. Biomarker data was generated among patients who consent to future studies and with tissue available in the biobank.
Data exclusions	No replication was done to the biomarker data due to limited patient specimens.
Replication	No replication was done to the biomarker data due to limited patient specimens.
Randomization	From November 2005 to September 2009 patients were randomized 1:1:1 to receive cetuximab, bevacizumab, or both of these biologic treatments in combination with either the mFOLFOX6 or FOLFIRI regimen. Thereafter, patients were randomized 1:1 to cetuximab or bevacizumab biologic treatment. At randomization, the primary physician indicated whether the treatment goal was palliative or potentially curative. Randomization was stratified by (1) chemotherapeutic regimen administered (mFOLFOX6; FOLFIRI), (2) receipt of prior adjuvant chemotherapy, and (3) prior pelvic radiation. Treatment assignments were generated according to randomly permuted blocks within strata. A fixed-block size of 9 was used prior to the study amendment that stopped enrollment into the double biologic group. Afterward, a block size of 6 was used.
Blinding	The CALGB 80405 study was an open-label, multicenter study. The treating physicians and patients were not blinded to the treatment assignment. During the biomarker generation, the laboratories which generate the biomarker data were blinded to the treatment assignments.

Reporting for specific materials, systems and methods

We require information from authors about some types of materials, experimental systems and methods used in many studies. Here, indicate whether each material, system or method listed is relevant to your study. If you are not sure if a list item applies to your research, read the appropriate section before selecting a response.

Materials & experimental systems

n/a	Involved in the study	
[x]	[ ]	Antibodies
[x]	[ ]	Eukaryotic cell lines
[x]	[ ]	Palaeontology and archaeology
[x]	[ ]	Animals and other organisms
[ ]	[x]	Human research participants
[ ]	[x]	Clinical data
[x]	[ ]	Dual use research of concern

Methods

n/a	Involved in the study	
[x]	[ ]	ChIP-seq
[x]	[ ]	Flow cytometry
[x]	[ ]	MRI-based neuroimaging

Human research participants

Policy information about studies involving human research participants

Population characteristics	Patients were enrolled at centers across the National Cancer Trials Network in the United States and Canada. Eligible patients had pathology-documented untreated locally advanced or metastatic colorectal cancer, although measurable disease (tumor that could be quantified) was not require. Patients had to be candidates for either mFOLFOX6 or FOLFIRI regimens without known central nervous system metastases or grade II or greater peripheral neuropathy. In addition, hypertension had to be well controlled (blood pressure <160/90 mm Hg with treatment) and there could be no concurrent congestive heart failure. Therapeutic anticoagulation was permitted as long as the patient was therapeutic on a stable dose of anticoagulant. Patients with a significant bleeding event within 6 months of enrollment or a gastrointestinal perforation within 12 months of enrollment were excluded unless the perforated bowel segment had been resected. Up to 6 months of prior adjuvant treatment had to have concluded at least 12 months before recurrence. Prior radiotherapy to 5040 cGy was allowed but could not have encompassed more than 25% of bone marrow. Patients were excluded if they had undergone major surgery within the last 4 weeks or minor surgery within the last 2 weeks. National Cancer Institute trials are required to capture and report data on race/ethnicity. Data for patient covariates were captured in the NCI-standardized format and entered at the time of patient registration usually by a clinical research associate at the treating institution. Race/ethnicity was determined by self-report.
Recruitment	Patients were 18 years or older with an Eastern Cooperative Oncology Group performance status of 0 to 1 and normal hepatic, renal, and hematologic laboratory values. In November 2008, KRAS wt (codons 12 and 13) became an eligibility criterion. Patients who included in this analysis will have to contribute sufficient tissue specimens; therefore, patients who did not consent to tissue collection or patients whose tissues have been exhausted prior to this analysis are not included in the analysis population. Patients who are included in this analysis are similar to patients who are excluded; except the variable describing whether the primary tumor is still in place. This is as expected since if the primary tumor is resected, the patient is more likely to contribute sufficient tissue specimens for laboratory analysis.
Ethics oversight	For each enrolling site, their IRB of record review and approve the protocol prior to patient enrollment.

Note that full information on the approval of the study protocol must also be provided in the manuscript.

Clinical data

Policy information about clinical studies

All manuscripts should comply with the ICMJEguidelines for publication of clinical research and a completedCONSORT checklist must be included with all submissions.

Clinical trial registration	NCT00265850
Study protocol	See https://clinicaltrials.gov/ct2/show/NCT00265850
Data collection	From September 2005 to March 2012, 3058 patients were preregistered or registered, and 2334 patients were randomized to 1 of 3 treatment groups at 396 study sites. Data quality was reviewed and audited by the Alliance Statistics and Data Center and by the study chairperson following Alliance policies. Data were released by the Alliance Data and Safety Monitoring Board in January 2014. Data was freeze in December 2015 for primary endpoint analysis. Biomarker data was generated in 2017 and 2018.
Outcomes	The primary endpoint of OS is defined as the time from registration to death. Patients who did not die were censored at last contact. The full description of the clinical trial primary and secondary endpoint and their descriptions can be found on the clinicalTrial.gov reporting. https://clinicaltrials.gov/ct2/show/NCT00265850?term=80405&draw=2&rank=3.
